# Anatomical variant of anteriorly extending mental foramen: A case report

**DOI:** 10.1002/ccr3.8341

**Published:** 2024-01-04

**Authors:** Ghassan Habash, Khaled R. Beshtawi, Soher Nagi Jayash

**Affiliations:** ^1^ Department of Dental Sciences, Faculty of Graduate Studies Arab American University Ramallah Palestine; ^2^ Palestinian Association of Dental Implantology (Palestinian Dental Association) Al‐Quds Palestine; ^3^ The Roslin Institute and Royal (Dick) School of Veterinary Studies University of Edinburgh Midlothian UK

**Keywords:** anatomical variation, case report, mental foramen, patient safety, superior anterior

## Abstract

**Key Clinical Message:**

By sharing this case, we aim to enhance the understanding of the mental foramen's intricate morphology, ultimately promoting safer and more successful surgical practices in the field of oral and maxillofacial surgery.

**Abstract:**

In surgical procedures near the mental foramen, preserving this vital structure and its contents is crucial. Surgical treatments, including procedures like implants, orthognathic surgery, and tooth extractions, can potentially lead to injuries of the mental nerve, resulting in sensory disturbances such as numbness or tingling in the lower lip and chin. This case report highlights an uncommon anterior extension of the mental foramen, posing a risk to the patient if unnoticed. Variations in this structure are possible, emphasizing the need for comprehensive three‐dimensional radiographic analysis before surgery to ensure patient safety. This report sheds light on the significance of identifying and understanding such variations to enhance the safety and precision of oral and maxillofacial interventions.

## Graphical abstract

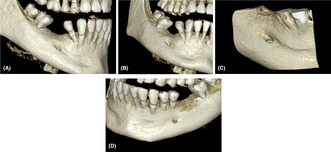

## 1 INTRODUCTION

The utilization of surgical treatment protocols, including endodontic retreatment, is frequently crucial, particularly for teeth concealed by complete restorations. These teeth are susceptible to fractures, often necessitating surgical endodontic retreatment. Such procedures come with inherent risks, both in terms of potential harm to noble structures and the anatomical variability of root structures.^1^ The mental foramen, a vital anatomical landmark in the mandible, plays a significant role in dental and maxillofacial surgery. It serves as a passageway for the mental nerve and vessels, making it crucial for various procedures such as nerve blocks and dental implant placements. Preservation of the mental foramen and its associated structures is imperative to prevent complications and ensure the success of surgical interventions.^2^ Despite its importance, anatomical variations in the mental foramen are well‐documented. These variations, though rare, can significantly impact surgical outcomes and patient safety. One such noteworthy variation is the anterior extension of the mental foramen, a condition seldom reported in the literature. Understanding and recognizing such variations are paramount to avoid inadvertent nerve damage and other complications during surgical procedures.^3^

The neurovascular bundles of the inferior mandibular canal emerge at the mental foramina (MF), which are the ending point apertures of the mental canals exiting the mandibular buccal sides.^4^ The chin, lower lip, and buccal gingiva in the anterior mandible bilaterally get sensory innervation and nourishment from the mental bundle, which travels via the mental foramen panoramic.^4^, ^5^ The mental canal inside the mandibular body may spread in numerous directions before reaching the MF i.e., superior, posterosuperior, labial, anterior, and posterior emergence profiles of the mental canal were reported.^6^, ^7^ The position of the mental foramen in both the horizontal and vertical planes, as well as its size and form, can also vary depending on race and gender.^8^ As a result, preoperative identification of the foramen is critical before numerous dental procedures.^7^, ^9^

In this context, we present a unique case report detailing an unusual anterior extension of the mental foramen. This case highlights the significance of detecting and comprehensively analyzing such anatomical variants before undertaking any surgical interventions in the mandibular region. Utilizing advanced radiographic techniques, including three‐dimensional (3D) imaging, is essential in identifying these variations, thereby ensuring precise surgical planning and safeguarding the patient's well‐being. This case report aims to contribute valuable insights into the field of maxillofacial anatomy, emphasizing the importance of meticulous preoperative assessment and awareness of rare anatomical variations.

## 2 CASE REPORT

**FIGURE 1:**
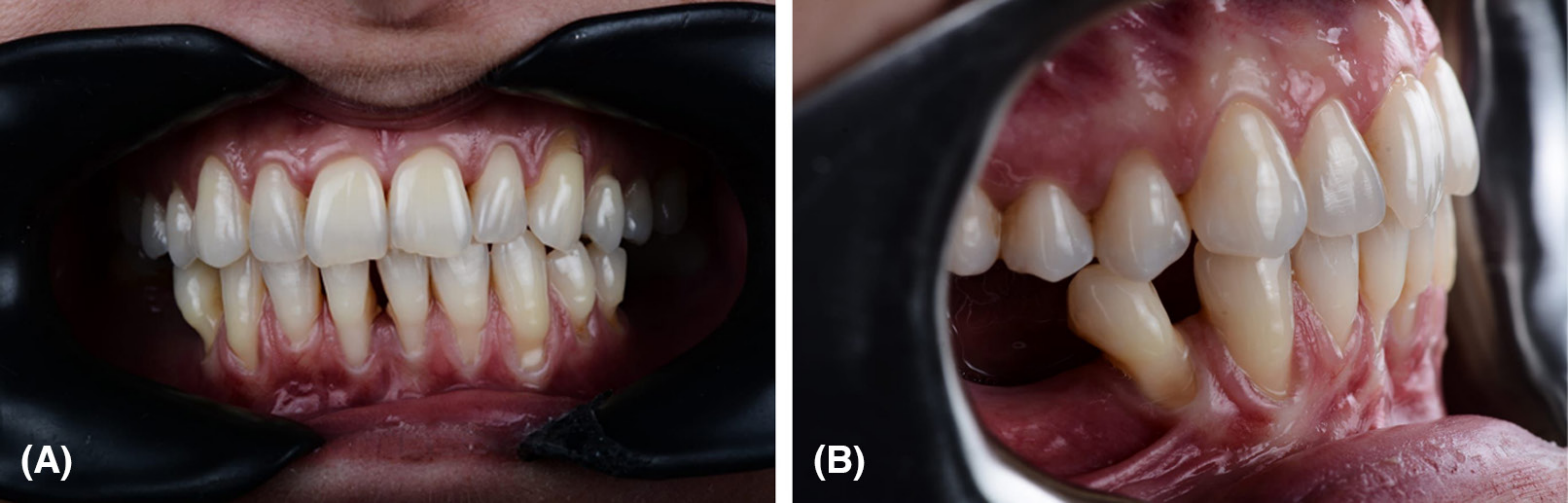
Clinical images of the patient showing the gingival recession (i.e., the main complain).

A 37‐year‐old, medically‐free, female patient presented at a private periodontology clinic based in Ramallah city, Palestine seeking to treat multiple gingival recessions. Comprehensive clinical examinations, including intraoral and extraoral assessments, to record any clinical or anatomical concerns were performed (Figure 1). A coronal advancement flap (region of teeth 42–32#) with two vertical releasing incisions and a connective tissue graft was suggested as the treatment plan.

### 2.1 Radiographical examinations

**FIGURE 2:**
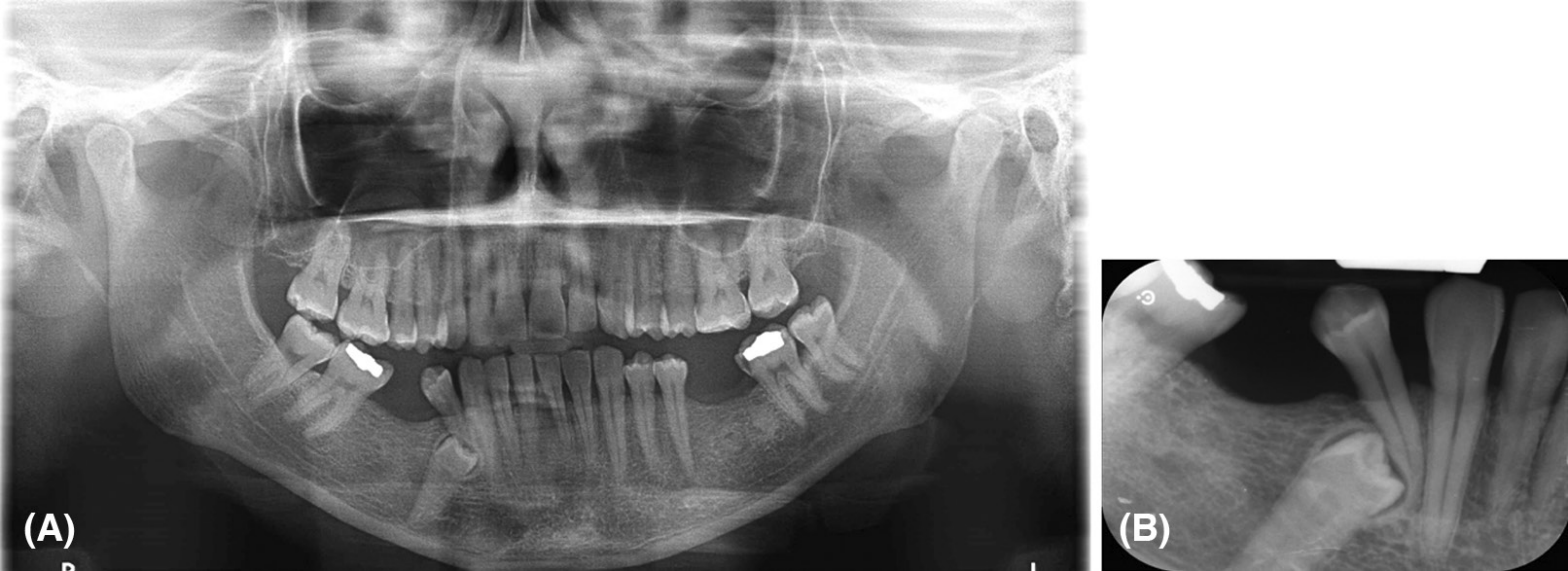
Panoramic (A) and periapical x‐ray (B) showing the impacted tooth #45 with no readily detectable mental foramen.

As part of the general examination procedures, panoramic and periapical radiographs were requested (Figure 2). Of the most important findings in the region of interest were the impacted tooth #45 and the adjacent radiolucent shadow inferior to the periapical region of teeth 42–44#. Moreover, MF were not clearly detectable. Advanced radiographic imaging i.e., cone‐beam computed tomography (CBCT) was requested to plan a surgical extraction of the tooth.

### 2.2 Data analysis

Radiographic images were analyzed by maxillofacial radiologist using specialized software to analyze the status of the impacted tooth and the adjacent structure and quantify the dimensions and spatial relationships of the anteriorly extending mental foramen.

The analysis of the case revealed a distinctive anatomical variation in the mandibular region. While panoramic and periapical radiographs initially indicated the presence of an impacted tooth (#45), the exact position and structure of the mental foramen were ambiguous (Figure 2). Subsequently, a CBCT scan was performed to gain a more detailed understanding of the mandibular anatomy.

**FIGURE 3:**
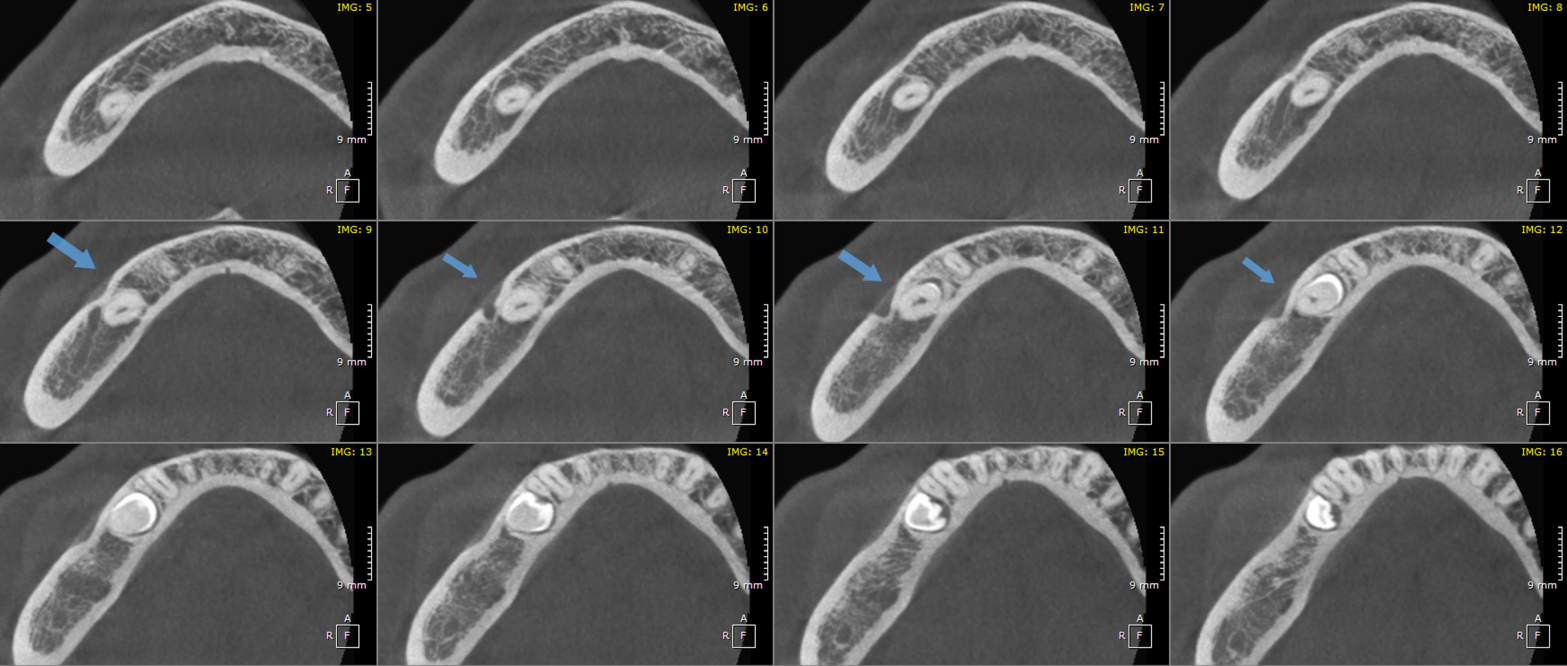
A series of CBCT axial slices (inferiorly → Superiorly) showing the unusual right‐side foramen's architecture with anterior extension (arrows).

**FIGURE 4:**
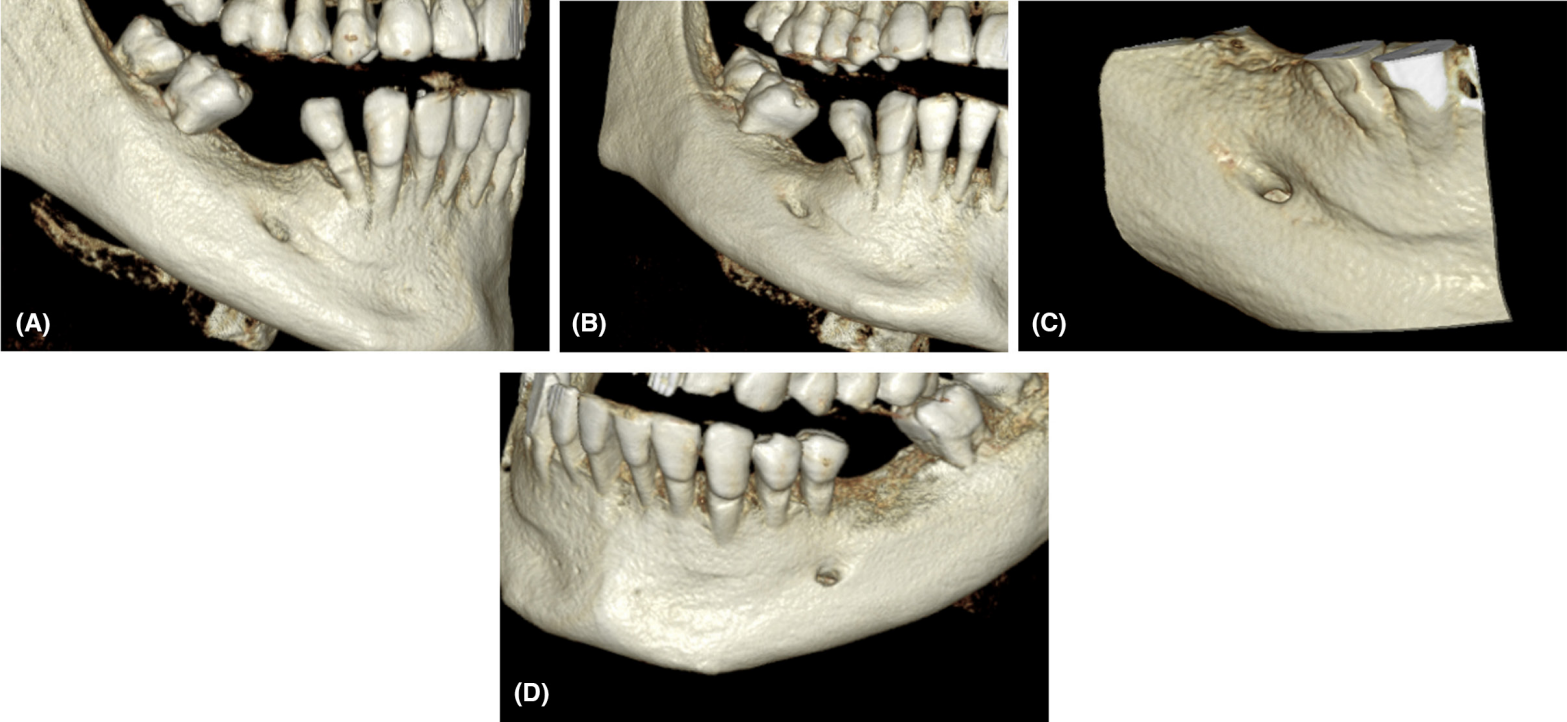
(A–C) Three‐dimensional (3D) model reconstructions of the right side of the patient showing the anterior extension of the mental foramen anteriorly. (D) 3D model of the left side showing usual foramen's architecture.

The CBCT images exhibited a clear depiction of the impacted tooth (#45) as well as the previously undetectable variation in the mental foramen's position and structure. This variation was meticulously studied, providing valuable insights into the unique morphology of the mental foramen in this particular case. The impacted tooth was lying directly anterior and lingual to the mental canal, with the mental canal showing an anterosuperiorly emergence profile. The bony region continuous with the foramen was showing a bony dentate extending anteriorly up to the periapical region of tooth # 44 (Figures 3 and 4). This extension which is identified by the indentation/groove of bone is highly suspicious for engulfing the track of one of the branches of the mental foramen.

### 2.3 Ethical consideration

Informed consent was obtained from the patient for inclusion in this case report.

## 3 DISCUSSION

The identification and understanding of anatomical variations are paramount in the practice of oral and maxillofacial surgery. Pre‐identification of the mental foramen structure before dental surgical procedures is vital to preserving the integrity of the neural vascular nourishment of the related anatomical structures.^7^, ^9^, ^10^ The identification of the mental foramen on conventional two‐dimensional radiographic modalities may not be reliable and the use of more sophisticated techniques would be necessary.^10^ In this case report, while traditional panoramic and periapical radiographs hinted at the presence of an impacted tooth (#45), they failed to provide clear insights into the position and structure of the mental foramen. This is particularly emphasized when trying to identify any possible anatomical variation in the structure, number, position, and most importantly emergence profiles.^10^ The subsequent use of CBCT proved instrumental in unraveling the complexity of the mandibular anatomy.^11^ The CBCT images revealed a previously undetectable variation in the mental foramen, emphasizing the limitations of conventional radiographic techniques in capturing intricate anatomical details. The detailed 3D visualization offered by CBCT not only confirmed the presence of the variation but also allowed for accurate measurements and characterization. In this case, we presented a unique variation involving the mental foramen, a critical anatomical landmark in the mandible. CBCT showed an anterior superior emergence profile of the mental canal, the canal extended and somehow dentate the buccal bone more anteriorly reaching.

Precise knowledge of the mental foramen's morphology is essential in various dental and maxillofacial interventions, including nerve blocks, dental implant placements, and oral surgeries.^12^ Failure to recognize such variations can lead to inadvertent nerve damage, postoperative complications, and compromised treatment outcomes.^13^

Moreover, this case underscores the importance of individualized treatment planning. Standard radiographs might not always suffice, especially in cases involving complex anatomical configurations. Utilizing advanced imaging techniques on a case‐by‐case basis ensures a more accurate assessment, allowing surgeons to tailor their approach according to the patient's unique anatomy.

## 4 CONCLUSION

In conclusion, this case report emphasizes the critical role of CBCT in uncovering rare anatomical variations. The meticulous analysis of such variations enhances our understanding of mandibular anatomy and, subsequently, improves the precision and safety of oral and maxillofacial surgical procedures. As clinicians, it is imperative to remain vigilant, employing advanced diagnostic tools when necessary, to provide optimal and personalized care for our patients.

## AUTHOR CONTRIBUTIONS

**Ghassan Habash:** Formal analysis; investigation; writing – review and editing. **Khaled R. Beshtawi:** Formal analysis; investigation; writing – original draft. **Soher Nagi Jayash:** Formal analysis; funding acquisition; writing – review and editing.

## FUNDING INFORMATION

We are grateful to the Biotechnology and Biological Sciences Research Council (BBSRC) for Institute Strategic Programme Grant Funding BB/J004316/1 to SNJ.

## CONFLICT OF INTEREST STATEMENT

The authors declare there are no competing interests.

## CONSENT

Written informed consent was obtained from the patient to publish this report in accordance with the journal's patient consent policy.

## Data Availability

All supporting data are available in manuscript.
