# Correction to Anatomical Variant of Anteriorly Extending Mental Foramen: A Case Report

**DOI:** 10.1002/ccr3.9562

**Published:** 2024-11-26

G. Habash, K. R. Beshtawi, S. N. Jayash, “Anatomical Variant of Anteriorly Extending Mental Foramen: A Case Report,” *Clinical Case Reports* 12, no. 1 (2024): e8341.

In the funding information section, the text “We are grateful to the Biotechnology and Biological Sciences Research Council (BBSRC) for Institute Strategic Programme Grant Funding BB/J004316/1 to SNJ” was incorrect. This should have read: “We are grateful to the Biotechnology and Biological Sciences Research Council (BBSRC) for Institute Strategic Programme Grant Funding (BBS/E/RL/230001C), which provided salary support to Soher Nagi Jayash during manuscript writing.”

We apologize for this error.

